# Predictors of nurses’ spiritual care competence: A replication study with Australian palliative care nurses

**DOI:** 10.1017/S1478951526102119

**Published:** 2026-04-28

**Authors:** Rita Mascio, Sandra Lynch, Jane Phillips, Megan Best

**Affiliations:** 1 Institute of Ethics and Society, University of Notre Dame, Broadway, NSW, Australia; 2 School of Nursing, Queensland University of Technology, Brisbane, QLD, Australia

**Keywords:** Nurse, spiritual care model, competence, replication, palliative care

## Abstract

**Objectives:**

There is a growing need to enhance healthcare providers’ spiritual care competence, including for people receiving palliative care. A preceding study of predictors of spiritual care competence in a general group of nurses found that more competent nurses rated significantly higher in spiritual training adequacy, frequency of spiritual care provision, and personal spirituality than other nurses; like the demographic variables of level of education, length of nursing experience, and sex, confidence and comfort in providing spiritual care were not related to spiritual care competence. The current study aimed to replicate these relationships in a sample of palliative care nurses. This sample also allowed the testing of a hypothesis that palliative care nurses will tend to subscribe to more competent understandings of spiritual care.

**Methods:**

Data were collected from a convenience sample of Australian palliative care nurses who completed an anonymous, online survey. The survey provided qualitative data about what spiritual care means for them and quantitative data regarding nurse characteristics. The qualitative data were used to create sub-groups of nurses based on their understanding of spiritual care and the quantitative data were used to construct a profile of nurse characteristics for each sub-group. The replication analysis determined whether a statistical difference in nurse characteristics existed across sub-groups. The hypothesis testing compared levels of spiritual care understanding across the general and palliative care samples of nurses.

**Results:**

While the results of the palliative care sample are largely concordant with those obtained in the general sample, the current study amends “training adequacy” as a predictor of spiritual care competence to “experience (whether on-the-job or training) in caring for the dying.” The study hypothesis was supported.

**Significance of results:**

The results can be used to assess and develop competence in spiritual care for palliative care nurses.

## Introduction

Spiritual care of patients nearing end-of-life is associated with many positive outcomes (Best et al. 2023). Hence, there is a growing call to enhance the capability of healthcare providers to routinely provide spiritual care (Selman et al. 2014, 2018). As nurses play a significant role in providing spiritual care to patients at end-of-life (Edwards et al. 2010), much research has focused on predictors of nurses’ spiritual care competence, defined here as the ability to assess and care for a patient’s spiritual needs (Green et al. 2019). According to this research, 5 particular characteristics have been commonly studied and can be regarded as predictors: comfort (e.g., Lundmark 2006), confidence (e.g., Jones et al. 2021) and frequency of spiritual care provision (Taylor et al. 1999), adequacy of training in spiritual care (e.g., Seid and Abdo 2022), and personal spirituality (e.g., Ross et al. 2018). While many studies found an association between these characteristics and spiritual care competence, they used *self-ratings* of spiritual care competence, which are generally flawed (Kruger and Dunning 1999).

In our preceding study (Mascio et al. 2024a) – hereon termed the “original study” – we re-examined the relationships between these commonly studied nurse characteristics and spiritual care competence, using a proxy for spiritual care competence comprising nurses’ understanding of spiritual care. Nurses understand spiritual care in 4 qualitatively different ways (described in Table 1), each yoked with different bundles of spiritual care behaviors, and arranged in order of increasing competence (Mascio et al. 2024b). Following Mascio et al. (2024b), the terms “understanding” and “model” will be used interchangeably to refer to a combination of the meaning and practice aspects of spiritual care. The original study found that nurses subscribing to Models C and D – the more competent models – rated significantly higher in spiritual care training adequacy, frequency of spiritual care provision, and personal spirituality than other nurses. Confidence and comfort in providing spiritual care were not significantly related to spiritual care competence and nor were demographic variables of level of nursing education, length of nursing experience, and sex.

**Table 1.** Models of spiritual care (Mascio et al. 2024b)

Model	Meaning of spiritual care	
	* Behaviors associated with spiritual care model	
**“A” Active management of the patient experience**	Spiritual care means deciding and performing actions that nurses unilaterally believe will bring patients peace and comfort, such as providing physical comfort, symptom control, or advice.	
	The nurses’ role is to assess patients’ situation and comfort levels based on what they themselves observe and to determine what actions are needed.	
	Nurses pilot or direct the experience of patients because patients are seen as unable to care for themselves (i.e., passive).	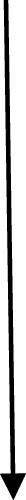
	Nurses are detached from patients; they minister to immediate specific needs (e.g., physical comfort) and try to manage physical surroundings to ensure a pleasant environment for patients.	
**“B” Responsive facilitation of patients’ wishes**	Spiritual care means eliciting/identifying from patients the emotional or spiritual needs/desires that have importance/value/meaning/comfort for them, and ensuring these needs are met to the best of their ability.	
	Nurses in this model aim to more actively engage patients by enquiring about and responding to patients’ overt expression of spiritual need.	
	Patients are viewed as “customers” with unique values and preferences, but they are distant or detached from nurses (i.e., not much “giving of self” by nurses).	
	Nurses prioritize giving service to patients; asking patients or family about the patient’s spiritual needs; performing concrete tasks/actions that meet explicitly stated needs.	*Increasing competence*
	Examples of actions that fulfill needs: answering questions related to end of life (e.g., explaining what will happen during the dying process); acting as coordinator, linking patients to family members or to services offered by unit (e.g., pastoral care); organizing religious activities requested by patients; facilitating the completion of final to-do things.	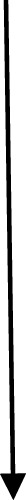
**“C” Accompany the patient on the dying journey**	Spiritual care means supporting patients in their final journey.	
	Nurses view patients as persons with unique backgrounds, unique approaches to death, needing individualized care.	
	Nurses aim to have patients share their thoughts and feelings about their situation so that they can understand how patients see their situation. Nurses want patients to know that they are not alone and to feel hope and security.	
	Nurses focus on personal involvement with patients. They are present with patients in a different way than in previous models: by giving time, attention, and “space” to patients, to allow conversation and discussion of patients’ thoughts and feelings to happen.	
	Establishing a trustful relationship with patients is important to enable emotional contact. Nurses stress the importance of listening during conversation, even when encounters are difficult.	
	Nurses encourage disclosure by the patient (but limits somewhat self-disclosure). Nurses aim to be flexible and ready to engage with patients whenever patients want to do so, and are comfortable with silence, with difficult emotions, and with the fact that they may not have answers to all of a patient’s questions.	
**“D” Empowering co-action with the patient**	Spiritual care is empowering co-action to walk alongside a fellow human being to move them along their unique, dying journey; collaborate/partner with the patient.	
	Nurses view patients as persons with unique backgrounds, approaches to death and needs, with whom nurses share a common humanity. Patients are perceived as having strengths, weaknesses, and personal resources and are willing and capable of managing the spiritual journey for themselves if they are supported, encouraged, and provided with resources they need in their journey.	
	Nurses see their role as a privileged one, co-operating with patients to uncover their power/strength in the face of pain and fear. The nurse strives to help patients transcend their situation, by encouraging patients to take responsibility and to be in charge of their experience, depending on individual capacity to do so.	
	Unlike Model C, in which the nurse is more of a listening board, the nurse in this model is more active and facilitates patients’ dying process, somewhat like a coach guiding a patient and asking questions to encourage reflection. Nurses try to understand patients as persons: their ideals, values, and experience of the situation, and use this information to guide conversation.	
	Like nurses in Model C, nurses know they don’t have all the answers to patients’ questions but are willing to help patients tackle these questions if patients so desire; nurses don’t try to avoid painful encounters, but neither does they try to solve the problem unilaterally. Nurses see themselves as a “resource” for patients and are willing to become personally involved by sharing of self and own humanity (e.g., emotions).	

The original study used a general sample of nurses in the United States who provided spiritual care to dying patients in a range of healthcare settings. That study noted a limitation that the external validity of the findings was unknown. The present study aims to address this limitation by assessing whether those findings can be replicated in a different sample comprising palliative care nurses who are more likely to encounter patients at the end of life requiring spiritual care.

### Replication in spiritual care research

A replication is the repetition of an original research study to verify or bolster confidence in its results (American Psychological Association 2018). One type of replication, a conceptual replication, intentionally varies aspects of a past study to test the degree to which the original results generalize to a new setting or population (Morrison 2022); that is, whether an effect extends to a different population given theoretical reasons to assume it will be weaker or stronger in a different group (Zwaan et al. 2018). Generalizability is important because disciplinary knowledge can be advanced only when empirical generalizations are present (Hubbard 2016). Replications make an original contribution to knowledge (Connelly 1986) by providing more information about the conditions under which the results occur (Cai et al. 2018), allowing the refinement and further development of a theory (Derksen and Morawski 2022). Within nursing, replications also contribute to building a body of evidence-based practice (Zadvinskis and Melnyk 2019). Consequently, replication is encouraged within all fields of science (Derksen et al. 2024) (e.g., journals have emphasized the benefits of replication in calls for papers, as a 2022 editorial in *Nature Communications* [Replication studies hold the key to generalization 2022] has done).

Despite the importance of replications being widely recognized (Feldman 1994), few replications are reported in nursing (Siedlecki 2024), psychology (Agnoli et al. 2021), and health literature generally (Sale and Mellor 2018). Commenting on the small number of replications in nursing, Feldman (1994) stated “if published research is a reflection of the development of a profession and its scholars, and replication is an integral contributor to knowledge building, we have a long way to go in developing nursing science” (p. 70). Decades later, the dearth of replications in nursing was again noted and another plea was again made for more replications to be conducted (Morin 2016).

The number of replications reported in the spiritual care literature also appears to be small. A computer search of the spiritual care literature (MEDLINE, CINAHL, Web of Science Core Collection, and Scopus databases from creation to 13.12.24 with “spiritual care” in the title and “replica*” anywhere in the article) identified 4 articles that explicitly claimed to be replications (Harris 1994; Clark and Heidenreich 1995; Lundmark 2006; Bar-Sela et al. 2019). At first glance, Feldman’s (1994) statement could also be applied to spiritual care research. We say “at first glance” because replication studies may be published but may not be explicitly identified as replications, as has been observed in other clinical contexts (Coiera and Tong 2021). The current study will be a conceptual replication of our previous study in a sample of palliative care nurses.

### Expected competence of this study’s sample

Besides investigating whether the original study results are held, this study’s use of a sample of palliative care nurses allows the testing of a hypothesis regarding their competence. The original study found that Model B was the most common spiritual care model in the general sample of nurses. In a sample of palliative care nurses, however, we expect that they will tend to subscribe to more competent models of spiritual care for 3 reasons. First, spiritual care is recognized as an essential part of palliative care (Best et al. 2020): the World Health Organization defines palliative care as a process involving “… early identification, correct assessment and treatment of pain and other problems, whether physical, psychosocial or spiritual” (World Health Organization 2020, para. 1).

Second, several systematic reviews of how nurses provide spiritual care to people at end-of-life describe behaviors and attributes that are concordant with Model C or D. For example, Gijsberts et al.’s (2019) systematic review found that spiritual care providers try to be present and to empower patients to work through issues to bring them peace. Batstone et al.’s (2020) systematic review found that nurses aim to provide holistic care that involves warmth and valuing of the patient; creates a spiritual nurse–patient connection through meaningful and fully present care; and facilitates the resolution of unmet spiritual needs using therapeutic communication skills. Edwards et al.’s (2010) systematic review found that spiritual care involves journeying with patients, actively listening to someone’s story for genuine understanding, respecting the uniqueness of each person, recognizing shared humanity, and partnering with patients to make shared decisions.

Third, palliative care nurses *in Australia* were chosen for this study. The palliative care context in Australia differs from that in the United States in that palliative patients in Australia have longer duration in palliative care (Jordan et al. 2020) as access to palliative care is not limited to the last 3 months of life and extensive in and out of hospital specialist palliative care services are available. A systematic review showed that nurses’ spiritual care provision is facilitated by a longer patient admission length and better nurse–patient relationship quality (Mascio et al. 2021) so the longer patients are receiving palliative care the more opportunity nurses have to develop trustful therapeutic relationships with patients which helps foster spiritual conversations.

## Methods

A cross-sectional survey design used in the original study was adapted for use with a group of 21 Australian nurses providing palliative care. A convenience sample of Australian palliative care nurses was recruited through emails sent to members of Palliative Care Nurses Australia, Palliative Care Victoria, Palliative Care NSW Inc., and Nurses Christian Fellowship Australia, inviting those nurses who provided spiritual care to complete the survey.

This study employed a nearly identical survey as the original study. The survey asked participants what spiritual care means for them and what they generally do to provide spiritual care; these open-ended questions were analyzed to identify the spiritual care model to which the nurses subscribed. The survey also asked for demographic details (nursing experience, palliative care experience, sex); how frequently they provided spiritual care; and their levels of agreement, using Likert scales, with statements relating to confidence and comfort providing spiritual care, personal spirituality, and adequacy of training in spiritual care. A question regarding goals in providing spiritual care was also asked; those results will be reported elsewhere.

### Data analysis

Data analysis proceeded in 2 stages. Stage 1 assessed the relationships between the nurse characteristics and spiritual care model. Stage 2 compared the palliative care and general nursing samples.

#### Stage 1. Relationships between nurse characteristics and spiritual care model

The procedure used in this stage was like that described in the original study, namely:

(i) Assigning respondents to Model A, B, C, or D groups based on an analysis of responses to the open-ended questions. This process satisfactorily coded all responses.

(ii) Calculating a statistical profile for each model group. Responses to questions regarding the predictor variables and demographic items were first assigned a numerical code, shown in Table 2, and medians calculated.

**Table 2.** Characteristics of participants (*N* = 21)

Characteristic	Code assigned to response during analysis	Number of nurses *n*
Nursing experience		
Less than 2 years	1	0
2–5 years	2	0
6–10 years	3	2
11–20 years	4	8
More than 20 years	5	11
Palliative care experience		
Less than 2 years	1	3
2–5 years	2	1
6–10 years	3	8
11–20 years	4	6
More than 20 years	5	3
Sex		
Male	1	0
Female	2	21
Frequency of spiritual care		
Sometimes	1	1
Often	2	15
Very often	3	5
		*Q1, Q2, Q3*^b^
Confidence^a^		4, 4, 4.5
Comfort^a^		4, 4, 5
Training adequacy^a^		2, 3, 3
Personal spirituality^a^		3, 4, 4

a Codes for Likert variables ranged from 1 = strongly disagree to 5 = strongly agree.

b *Q*1, *Q*2, *Q*3 denote first, second, and third quartiles.

(iii) Statistical testing of differences between model groups.

Regarding step (iii), the original study utilized the Kruskal–Wallis test to assess for differences between each of the 4 groups because the sample size was quite large (*N* = 66). In the current study, however, it eventuated that too few respondents were assigned to Model A and Model D cells for the Kruskal–Wallis test to be used; this test requires at least 5 respondents in each group (Newbold 1988). To overcome this difficulty, Model A and B groups were combined, as were Model C and D groups, to form 2 larger groups. This allowed the Mann–Whitney test to be used for the statistical comparison of the resulting 2 groups. Combining groups in this way still allowed us to test whether the significant findings of the original study held.

#### Stage 2. Comparison of general and palliative care samples

This stage tested the hypothesis that nurses in the palliative care sample were more likely to subscribe to more competent models of spiritual care than the general sample. The overall level of spiritual care competence in a sample was operationalized as the median spiritual care model of the sample. Because the spiritual care models could be ranked in order of competence (Mascio et al. 2024b), Models A, B, C, and D were assigned numeric codes of 1, 2, 3, and 4, respectively. The medians of this variable were calculated for both samples, and the Mann–Whitney test assessed differences in medians.

This stage also included an exploratory supplementary analysis to examine whether nurse characteristics (comfort, confidence, experience, etc.) differed between the general and palliative samples. The median values of these characteristics were first calculated for each sample and then Mann–Whitney tests compared nurse characteristics across both samples.

## Results

Participants in the sample (*N* = 21) were all female, had 6 or more years of nursing experience, and ranged in palliative care experience (up to 20+ years). Table 2 shows the sample characteristics. Table 3 shows the numbers of participants in each spiritual care model group and example responses to the open-ended questions.

**Table 3.** Coding of spiritual care model in the palliative sample

Model	Number of participants	Sample quotes^a^
A	1	*Read to patients, positive words, providing a bright, positive atmosphere.*
B	7	*Ask patients what they need for comfort/peace, offer to pray with them or to have pastoral or psychologist visit.*
C	9	*Mainly listen to what the patient has to say, show support by being present, not rushing things, prioritise conversation with patient over other things.*
D	4	*Coach the patient to help them understand and resolve their own situation, ask open-ended questions, active listening, being non-judgemental and empathetic.*

a Minor spelling errors have been corrected.

### Results of stage 1 analysis

Table 4 shows the statistical profiles of each spiritual care model group within the palliative care sample. The Model C and D combined group was associated with higher levels of palliative care experience (*Z* = 2.80, *p* = 0.003), greater frequency of spiritual care provision (*Z* = 2.20, *p* = 0.03), and higher personal spirituality (*Z* = 2.21, *p* = 0.02) than the Model A and B combined group. However, the combined groups did not differ significantly from each other in training adequacy (*Z* = 1.66, *p* = 0.12), confidence (*Z* = 0.09, *p* = 0.59), comfort (*Z* = 1.47, *p* = 0.11), or nursing experience (*Z* = 1.42, *p* = 0.10).

**Table 4.** Statistical profiles of spiritual care model groups within the palliative sample

	Spiritual care model				Test statistic^a^	*p*-value^b^
	A	B	C	D		
	(*n* = 1)	(*n* = 7)	(*n* = 9)	(*n* = 4)		
Median frequency	1	2	2	2	2.20	0.03*
Median spirituality	3	4	4	4	2.21	0.02*
Median training adequacy	3	2	3	3	1.66	0.12
Median confidence	4	4	4	4	0.09	0.59
Median comfort	3	4	4	4	1.47	0.11
Median nursing experience	4	4	5	4.5	1.42	0.10
Median palliative care experience	1	3	4	4	2.80	0.003*
Median sex^c^	2	2	2	2	-	-

a Mann–Whitney tests to assess difference between Model B and Model C showed similar significant and insignificant results.

b Bonferroni corrections have not been applied as results are subject to further testing (Armstrong 2014). Significant differences are denoted with asterisk.

c Statistical tests across sex could not be conducted because the whole sample was female.

### Results of stage 2 analysis

**Figure 1.**
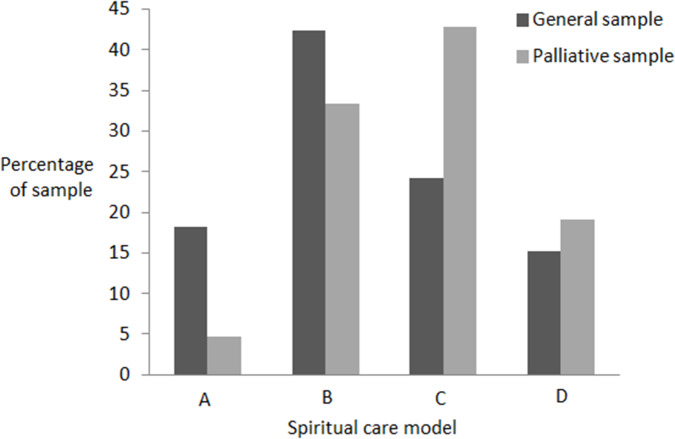
Distribution of models in the original and replications samples.

Figure 1 shows the distribution of models in the general and palliative care samples. In the general sample, the most common model was Model B and 61% of participants subscribed to Model A or B. In the palliative care sample, the most common model was Model C and 38% of participants subscribed to Model A or B.

Table 5 shows the median spiritual care model of both samples. The palliative care sample subscribed to more competent models of spiritual care than the general sample of nurses (*Z* = 1.78, *p* = 0.04), as hypothesized. This table also shows that frequency of spiritual care (*Z* = 3.12, *p* < 0.001), personal spirituality (*Z* = 3.95, *p* < 0.001), confidence (*Z* = 4.16, *p* < 0.001), and comfort (*Z* = 4.13, *p* < 0.001) were significantly higher in the palliative care sample than in the general sample. However, training adequacy, nursing experience, and sex did not differ significantly between the samples.

**Table 5.** Statistical profiles of the original and replication samples

Variable	General sample	Palliative sample	Test statistic	*p*-value^a^
	(*n* = 66)	(*n* = 21)		
Median spiritual care model^b^	2	3	1.78	0.04*
Median frequency	1	2	3.12	<0.001*
Median spirituality	3	4	3.95	<0.001*
Median training adequacy	2.5	3	1.09	0.15
Median confidence	3	4	4.16	<0.001*
Median comfort	3	4	4.13	<0.001*
Median nursing experience	4	5	1.58	0.06
Mode sex	2	2	0.62	0.54

a Significant differences (*p* < 0.05) are denoted with an asterisk.

b Spiritual care models A, B, C, and D were coded as 1, 2, 3, and 4, respectively.

## Discussion

This study assessed whether the predictors of spiritual care competence identified in Mascio et al. (2024a) could be found among palliative care nurses. Comparison of the results from the original and replication studies has implications for the assessment and development of spiritual care competence.

The results regarding the predictors of spiritual care competence are quite similar in the original and replication studies. Let us consider first results relating to frequency of spiritual care provision. Frequency was significantly higher among palliative care nurses than in a general group of nurses; this accords with the literature that finds that palliative care nurses provide spiritual care more frequently than nurses practicing in other clinical areas (Taylor et al. 2023). In addition, consistent with the general group of nurses (Mascio et al. 2024a), frequency was significantly associated with more competent models among palliative care nurses. These results are expected because palliative care nurses have more contact with patients who are dying, a group known to have an increased incidence of spiritual need (Egan et al. 2017), and therefore palliative care nurses have more opportunities to enhance competence in spiritual care.

The results regarding personal spirituality paralleled those relating to frequency: spirituality was higher among palliative care nurses than among a general group of nurses. Again, this result aligns with other studies that compare the spirituality of palliative/hospice nurses with nurses in different departments (e.g., Taylor et al. 1999; Ronaldson et al. 2012). Consistent with the general group of nurses, spirituality was also associated with more competent models among palliative care nurses. The regular relationship between spiritual care frequency and spirituality – across models within a general group of nurses and within a group of palliative care nurses, and between the 2 cohorts – suggests that these 2 factors play consistent and strong roles in the development of competence in spiritual care. This suggestion aligns with Taylor et al.’s (1999) finding that frequency and personal spirituality are both highly correlated (*r*(*709*) = 0.58, *p* < 0.001; *r*(*709*) = 0.47, *p* < 0.001) respectively) with ability to provide spiritual care among oncology and hospice nurses.

Confidence and comfort did not differ across spiritual care models in the original and replication studies. These results are discrepant with a recent systematic review (Mascio et al. 2021), which consistently found that these characteristics facilitated spiritual care, and their lack was a barrier. A possible reason for this discrepancy could be that the empirically based original and replication studies and the systematic review investigated different types of nurse behaviors: the former empirical studies examine the relationships between confidence/comfort and *specific bundles* of behaviors as delineated by the spiritual care models (i.e., Model A versus Model B versus Model C versus Model D behaviors) while the latter review study (Mascio et al. 2021) examined the relationship between confidence/comfort and *any form* of spiritual care behavior, rather than specific bundles of behavior. Additionally, both empirical studies excluded nurses who did not provide spiritual care; perhaps if they were included, an effect of comfort and confidence may have been observed. In other words, confidence and comfort may influence *whether or not* a nurse provides spiritual care but may not impact *how* they provide that spiritual care. Further research is needed to confirm this conjecture.

The results regarding confidence and comfort did accord, however, with the finding that these factors are not useful indicators of performance in a task because individuals can overestimate their capabilities through overconfidence (Chi 2006). In reality, most individuals maintain a sufficiently stable and satisfactory, although not necessarily expert, level of performance with which they are comfortable (Ericsson and Ferrari 2002). Nonetheless, overall levels of confidence and comfort among the palliative care nurses *were* higher than those among a general group of nurses. While this result may seem unusual at first, it could be that these measures tap into nurses’ confidence and comfort in relating to patients at end-of-life generally. Since palliative care considers people’s spiritual needs, and these nurses have more exposure to dying patients, it is plausible that palliative care nurses felt more confident/comfortable relating to patients facing death. Further research is needed to discover the constituent dimensions of comfort and confidence in the spiritual care task.

The results relating to experience are ambivalent, depending on the type of experience examined. The length of nursing experience did not differ significantly across spiritual care models either within the general group of nurses or among palliative care nurses, and even though it was higher in the latter compared to the former, this result did not reach significance. This lack of relationship between nursing experience and competence aligns with previous findings that longevity in a profession alone does not automatically guarantee expertise (Ericsson et al. 2007).

However, the result that palliative care experience was significantly associated with more competent models does not initially seem to align with the view that experience bears little relationship to performance (Ericsson et al. 2007). However, palliative care experience differs from general nursing experience, as it is focuses on caring for individuals with life-limiting illnesses, with spiritual care being an integral component of effective palliative care (Best et al. 2020). Hence, palliative care experience could be a type of on-the-job training that facilitates spiritual care work, a surmise that is supported by the finding that the palliative care nurses tended to subscribe to more competent models of spiritual care than the general group of nurses.

The surmise regarding on-the-job training is complemented by results relating to spiritual care training adequacy. In the original study involving a general group of nurses, nurses subscribing to Models C and D rated significantly higher in spiritual care training adequacy than other nurses. But among palliative care nurses, training adequacy did not differ significantly across models. In other words, a general group of nurses may benefit from spiritual care training but palliative care nurses may benefit from on-the-job training.

One curious result is that in both the general group of nurses and palliative care nurses the number of nurses subscribing to Model D was approximately half the number subscribing to Model C. In the general group, the ratio was 16:10 and among palliative care nurses this ratio was 9:4. This might suggest that moving from the competence level of Model C to that of Model D does not require skill in relating to dying patients and that the hurdle is the same in both groups of nurses. This begs the question of what that hurdle could be. One possibility lies in the observation that a difference between C and D models is the directivity of the nurse in spiritual conversations (Mascio et al. 2024b), which is the influence of the nurse, relative to the patient, in specifying the patient’s spiritual needs and how those needs are to be fulfilled (Mascio et al. 2024b). In Model C, nurses are less directive in that they follow the patient’s lead in conversation, whereas in Model D nurses are more directive in that they actively probe and guide the conversation. It could be that this capability – of being more directive and of probing and guiding conversation – is difficult to develop and may require more specific training. This is something that future research could explore.

While most of the palliative care nurses subscribed to Model C or D, just over a third of nurses (38%) subscribed to Model A or B, which seems consistent with a study by Keall et al. (2014). In that study of 20 Australian palliative care nurses from diverse health settings, approximately one third (i.e., 7) feared what they may uncover in spiritual conversations with patients and their ability to manage their response. Nurses who subscribe to Model C or D are not afraid of not knowing an answer to a question (Mascio et al. 2024b), which suggests that those 7 nurses in Keall et al.’s (2014) sample subscribed to Model A or B. Further research is needed to determine the actual proportions of palliative care nurses who subscribe to the different models as this will be one indicator of the general quality of spiritual care provided; if Model D is the “ideal” model of spiritual care (Mascio et al. 2024b), significant departures from the ideal may lead to poor care for patients who are unable to articulate their needs, which could exacerbate their suffering (Best et al. 2023).

Overall, the results from the original and replication studies suggest that (a) personal spirituality, frequency of spiritual care provision, and some kind of experience in caring for dying (whether on-the-job or via the classroom training) are helpful in developing competence in spiritual care, whereas general nursing experience is not, and (b) comfort and confidence in providing spiritual care are not related to spiritual care competence. While the replication results are largely concordant with those of the original study, the current study refines “training adequacy” as a predictor to “experience (whether on-the-job or training) in caring for the dying.”

### Limitations and future research

The current study’s use of a nearly identical survey as the original study allowed a direct comparison of predictors of spiritual care competence between a general group of nurses and palliative care nurses. However, this strength needs to be balanced with several method-related factors that compromise the representativeness of data and interpretation of results. The use of a convenience sample of palliative care nurses means that the representativeness of respondents is unknown. In addition, single-item measures were used to estimate the predictor variables to minimize nurse fatigue and consequent low response rates (Bethel et al. 2021), but these measures may have compromised criterion validity. The sample size necessitated combining the Model A and B groups, as well as the Model C and D groups, to enable statistical tests to be conducted. Furthermore, even though the ordinal data elicited via the closed questions necessitated the use of the Mann–Whitney test, this test has less power to detect differences when they actually occur (Newbold 1988). Future research should examine a larger more representative sample using multi-item estimates of the predictor variables and more powerful statistical tests to enhance the external validity of findings.

## Conclusion

In an earlier study of predictors of spiritual care competence in a general group of American nurses (Mascio et al. 2024a), those subscribing to more competent models of spiritual care tended to rate significantly higher in training adequacy, frequency of spiritual care provision, and personal spirituality than other nurses. However, like demographic variables of education level, length of nursing experience, and sex, confidence and comfort in providing spiritual care were not related to spiritual care competence. The current study aimed to replicate these relationships in a sample of Australian palliative care nurses. While the results are largely concordant with the original results, the current study amends “training adequacy” as a predictor of spiritual care competence to “experience (whether on-the-job or training) in caring for the dying.” As well, the study supported the hypothesis that palliative care nurses tend to subscribe to more competent understandings of spiritual care. The results can be used to assess and develop competence in spiritual care.

## References

[ref1] Agnoli F, Fraser H, Singleton Thorn F, et al. (2021) Australian and Italian psychologists’ view of replication. *Advances in Methods and Practices in Psychological Science* 4(3). doi:10.1177/25152459211039218

[ref2] American Psychological Association (2018). Replication. https://dictionary.apa.org/replication (accessed 16 December 2024).

[ref3] Armstrong RA (2014) When to use the Bonferroni correction. *Ophthalmic & Physiological Optics* 34(5), 502–508. doi:10.1111/opo.12131 24697967

[ref4] Bar-Sela G, Schultz MJ, Elshamy K, et al. (2019) Human development index and its association with staff spiritual care provision: A Middle Eastern oncology study. *Supportive Care in Cancer* 27(9), 3601–3610. doi:10.1007/s00520-019-04733-0 30895381

[ref5] Batstone E, Bailey C and Hallett N (2020) Spiritual care provision to end‐of‐life patients: A systematic literature review. *Journal of Clinical Nursing* 29(19-20), 3609–3624. doi:10.1111/jocn.15411 32645236

[ref6] Best M, Leget C, Goodhead A, et al. (2020) An EAPC white paper on multi-disciplinary education for spiritual care in palliative care. *BMC Palliative Care* 19(1), 9. doi:10.1186/s12904-019-0508-4 31941486 PMC6964109

[ref7] Best MC, Vivat B and Gijsberts M-J (2023) Spiritual care in palliative care. *Religions* 14(3), 320. doi:10.3390/rel14030320

[ref8] Bethel C, Rainbow JG and Dudding KM (2021) Recruiting nurses via social media for survey studies. *Nursing Research* 70(3), 231–235. doi:10.1097/NNR.0000000000000482 33060416

[ref9] Cai J, Morris A, Hohensee C, et al. (2018) The role of replication studies in educational research. *Journal for Research in Mathematics Education* 49(1), 2–8. doi:10.5951/jresematheduc.49.1.0002

[ref10] Chi MTH (2006) Two approaches to the study of experts’ characteristics. In Charness N, Ericsson KA, Feltovich PJ and Hoffman RR (eds), *The Cambridge Handbook of Expertise and Expert Performance*. Cambridge: Cambridge University Press, 21–30.

[ref11] Clark C and Heidenreich T (1995) Spiritual care for the critically ill. *American Journal of Critical Care* 4(1), 77–81. doi:10.4037/ajcc1995.4.1.77 7894561

[ref12] Coiera E and Tong HL (2021) Replication studies in the clinical decision support literature–frequency, fidelity, and impact. *Journal of the American Medical Informatics Association* 28(9), 1815–1825. doi:10.1093/jamia/ocab049 34226931 PMC8363796

[ref13] Connelly CE (1986) Replication research in nursing. *International Journal of Nursing Studies* 23(1), 71–77. doi:10.1016/0020-7489(86)90039-8 3632952

[ref14] Derksen M, Meirmans S, Brenninkmeijer J, et al. (2024) Replication studies in the Netherlands: Lessons learned and recommendations for funders, publishers and editors, and universities. *Accountability in Research*, 1–19. doi:10.1080/08989621.2024.2383349 39135508

[ref15] Derksen M and Morawski J (2022) Kinds of replication: Examining the meanings of “conceptual replication” and “direct replication.” *Perspectives on Psychological Science* 17(5), 1490–1505. doi:10.1177/17456916211041116 35245130 PMC9442273

[ref16] Edwards A, Pang N, Shiu V, et al. (2010) Review: The understanding of spirituality and the potential role of spiritual care in end-of-life and palliative care: a meta-study of qualitative research. *Palliative Medicine* 24(8), 753–770. doi:10.1177/0269216310375860 20659977

[ref17] Egan R, MacLeod R, Jaye C, et al. (2017) Spiritual beliefs, practices, and needs at the end of life: Results from a New Zealand national hospice study. *Palliative & Supportive Care* 15(2), 223–230. doi:10.1017/S147895151600064X 27572901

[ref18] Ericsson KA and Ferrari M (2002) Attaining excellence through deliberate practice: insights from the study of expert performance. In Ferrari M (ed), *The Pursuit of Excellence through Education*. London: Routledge, 21–56.

[ref19] Ericsson KA, Whyte TJ and Ward P (2007) Expert performance in nursing: reviewing research on expertise in nursing within the framework of the expert-performance approach. *Advances in Nursing Science* 30(1), E58–E71. doi:10.1097/00012272-200701000-00014 17299276

[ref20] Feldman HR (1994) Developing nursing science: A case for replication. *Journal of Professional Nursing* 10(2), 70–70. doi:10.1016/8755-7223(94)90065-5 8027481

[ref21] Gijsberts M-J, Liefbroer AI, Otten R, et al. (2019) Spiritual care in palliative care: A systematic review of the recent European literature. *Medical Sciences* 7(2), 1–21. doi:10.3390/medsci7020025 PMC6409788 30736416

[ref22] Green A, Kim-Godwin YS and Jones CW (2019) Perceptions of spiritual care education, competence, and barriers in providing spiritual care among registered nurses. *Journal of Holistic Nursing* 38(1), 41–51. doi:10.1177/0898010119885266 31690159

[ref23] Harris K (1994) *Nurses’ Attitudes Towards Providing Spiritual Care: A Replication Study*. Master’s thesis, Bellarmine College.

[ref24] Hubbard RT (2016) *Corrupt Research: The Case for Reconceptualizing Empirical Management and Social Science*. Los Angeles, CA: SAGE Publications, Inc.

[ref25] Jones KF, Paal P, Symons X, et al. (2021) The content, teaching methods and effectiveness of spiritual care training for healthcare professionals: A mixed-methods systematic review. *Journal of Pain and Symptom Management* 62(3), e261–e278. doi:10.1016/j.jpainsymman.2021.03.013 33757893

[ref26] Jordan RI, Allsop MJ, ElMokhallalati Y, et al. (2020) Duration of palliative care before death in international routine practice: A systematic review and meta-analysis. *BMC Medicine* 18(1), 368–368. doi:10.1186/s12916-020-01829-x 33239021 PMC7690105

[ref27] Keall R, Clayton JM and Butow P (2014) How do Australian palliative care nurses address existential and spiritual concerns? Facilitators, barriers and strategies. *Journal of Clinical Nursing* 23(21-22), 3197–3205. doi:10.1111/jocn.12566 25453124

[ref28] Kruger J and Dunning D (1999) Unskilled and unaware of it: How difficulties in recognizing one’s own incompetence lead to inflated self-assessments. *Journal of Personality and Social Psychology* 77(6), 1121–1134. doi:10.1037/0022-3514.77.6.1121 10626367

[ref29] Lundmark M (2006) Attitudes to spiritual care among nursing staff in a Swedish oncology clinic. *Journal of Clinical Nursing* 15(7), 863–874. doi:10.1111/j.1365-2702.2006.01189.x 16879379

[ref30] Mascio R, Best M, Lynch S, et al. (2021) Factors influencing nurse spiritual care practices at the end of life: A systematic review. *Palliative & Supportive Care*, 1–19. doi:10.1017/S1478951521001851 34872626

[ref31] Mascio R, Lynch S, Phillips JL, et al. (2024a) Nurses’ models of spiritual care: Predictors of spiritual care competence. *Palliative & Supportive Care*, 1–8. doi:10.1017/S1478951524000750 39534942

[ref32] Mascio R, Lynch S, Phillips JL, et al. (2024b) Nurses’ models of spiritual care: A cross-sectional survey of American nurses. *Palliative & Supportive Care*, 1–11. doi:10.1017/S1478951523000676 37435660

[ref33] Morin KH (2016) Replication: Needed now more than ever. *Journal of Nursing Education* 55(8), 423–424. doi:10.3928/01484834-20160715-01 27459427

[ref34] Morrison K (2022) Conceptual replications, research, and the “what works” agenda in education. *Educational Research and Evaluation* 27(1-2), 35–60. doi:10.1080/13803611.2021.2022314

[ref35] Newbold P (1988) *Statistics for Business and Economics*. Englewood Cliffs, NJ: Prentice-Hall International.

[ref36] Replication studies hold the key to generalization (2022) Editorial. *Nature Communications* 13(1), 7004–7004. doi:10.1038/s41467-022-34748-x PMC9668916 36385242

[ref37] Ronaldson S, Hayes L, Aggar C, et al. (2012) Spirituality and spiritual caring: Nurses’ perspectives and practice in palliative and acute care environments. *Journal of Clinical Nursing* 21(15‐16), 2126–2135. doi:10.1111/j.1365-2702.2012.04180.x 22788554

[ref38] Ross L, McSherry W, Giske T, et al. (2018) Nursing and midwifery students’ perceptions of spirituality, spiritual care, and spiritual care competency: A prospective, longitudinal, correlational European study. *Nurse Education Today* 67, 64–71. doi:10.1016/j.nedt.2018.05.002 29763841

[ref39] Sale C and Mellor D (2018) A call for replication studies in Nutrition and Health. *Nutrition and Health* 24(4), 201–201. doi:10.1177/0260106018817675

[ref40] Seid K and Abdo A (2022) Nurse’s spiritual care competence in Ethiopia: A multicenter cross-sectional study. *PLoS One* 17(3), e0265205–e0265205. doi:10.1371/journal.pone.0265205 35271676 PMC8912899

[ref41] Selman LBAMP, Young TB, Vermandere MMD, et al. (2014) Research priorities in spiritual care: An international survey of palliative care researchers and clinicians. *Journal of Pain and Symptom Management* 48(4), 518–531. doi:10.1016/j.jpainsymman.2013.10.020 24680625

[ref42] Selman LE, Brighton LJ, Sinclair S, et al. (2018) Patients’ and caregivers’ needs, experiences, preferences and research priorities in spiritual care: A focus group study across nine countries. *Palliative Medicine* 32(1), 216–230. doi:10.1177/0269216317734954 29020846 PMC5758929

[ref43] Siedlecki SL (2024) Replication research and metascience: Prerequisite for evidence-based nursing practice. *Clinical Nurse Specialist* 38(2), 69–71. doi:10.1097/NUR.0000000000000804 38364065

[ref44] Taylor EJ, Highfield MF and Amenta M (1999) Predictors of oncology and hospice nurses’ spiritual care perspectives and practices. *Applied Nursing Research* 12(1), 30–37. doi:10.1016/S0897-1897(99)80156-6 10048239

[ref45] Taylor EJ, Pariñas S, Mamier I, et al. (2023) Frequency of nurse‐provided spiritual care: An international comparison. *Journal of Clinical Nursing* 32(3-4), 597–609. doi:10.1111/jocn.16497 36039033 PMC10087347

[ref46] World Health Organization (2020) WHO definition of palliative care. https://www.who.int/news-room/fact—sheets/detail/palliative—care#:∼:text=Each%20year%2C%20an%20estimated%2056.8,in%20need%20of%20palliative%20care. (accessed 5 January 2026d).

[ref47] Zadvinskis IM and Melnyk BM (2019) Making a case for replication studies and reproducibility to strengthen evidence‐based practice. *Worldviews on Evidence-Based Nursing* 16(1), 2–3. doi:10.1111/wvn.12349 30758132

[ref48] Zwaan RA, Etz A, Lucas RE, et al. (2018) Making replication mainstream. *Behavioral and Brain Sciences* 41, 1–e120. doi:10.1017/S0140525X17001972 29065933

